# C-Reactive Protein and Its Relationship with Pain in Patients with Advanced Cancer Cachexia: Secondary Cross-Sectional Analysis of a Multicenter Prospective Cohort Study

**DOI:** 10.1089/pmr.2021.0004

**Published:** 2021-05-05

**Authors:** Koji Amano, Hiroto Ishiki, Tomofumi Miura, Isseki Maeda, Yutaka Hatano, Shunsuke Oyamada, Naosuke Yokomichi, Keita Tagami, Takuya Odagiri, Tetsuya Ito, Mika Baba, Tatsuya Morita, Masanori Mori

**Affiliations:** ^1^ Department of Palliative Medicine, National Cancer Center Hospital, Chuo-ku, Tokyo, Japan.; ^2^ Department of Palliative and Supportive Medicine, Graduate School of Medicine, Aichi Medical University, Nagakute, Aichi, Japan.; ^3^ Department of Palliative Medicine, National Cancer Center Hospital East, Kashiwa, Chiba, Japan.; ^4^ Department of Palliative Care, Senri-chuo Hospital, Toyonaka, Osaka, Japan.; ^5^ Department of Palliative Care, Daini Kyoritsu Hospital, Kawanishi, Hyogo, Japan.; ^6^ Department of Biostatistics, JORTC Data Center, Arakawa-ku, Tokyo, Japan.; ^7^ Department of Palliative and Supportive Care, Seirei Mikatahara General Hospital, Hamamatsu, Shizuoka, Japan.; ^8^ Department of Palliative Medicine, Tohoku University Graduate School of Medicine, Sendai, Miyagi, Japan.; ^9^ Department of Palliative Care, Komaki City Hospital, Komaki, Aichi, Japan.; ^10^ Department of Palliative Care, Japanese Red Cross Medical Center, Shibuya-ku, Tokyo, Japan.; ^11^ Department of Palliative Medicine, Suita Tokushukai Hospital, Suita, Osaka, Japan.

**Keywords:** advanced cancer, cancer cachexia, C-reactive protein, pain, palliative care

## Abstract

***Background:*** Limited information is available on the relationship between C-reactive protein (CRP) levels and pain in advanced cancer.

***Objectives:*** To investigate the relationship between serum levels of CRP and subtypes of pain.

***Design:*** A secondary cross-sectional analysis of a prospective cohort study.

***Setting/Subjects:*** Patients with advanced cancer admitted to 23 palliative care units in Japan.

***Measurements:*** Patients rated the severity of pain on the numerical rating scale (NRS) and physicians evaluated pain on the integrated palliative care outcome scale (IPOS). Physicians assessed neuropathic pain and breakthrough pain based on their presence or absence. Patients were divided into four groups according to CRP levels. Comparisons were performed using the Kruskal–Wallis test or chi-squared test. To evaluate the relationship between CRP and subtypes of pain, adjusted odds ratios (ORs) and 95% confidence intervals (CIs) in logistic models were calculated.

***Results:*** We divided 1513 patients into four groups: low CRP (*n* = 234), moderate CRP (*n* = 513), high CRP (*n* = 352), and very high CRP (*n* = 414). Spearman's correlation coefficient between CRP and pain NRS and that between CRP and pain IPOS were 0.15 (*p* < 0.001) and 0.16 (*p* < 0.001), respectively. In the models of pain NRS and pain IPOS, significantly higher adjusted ORs than in the low CRP group were observed in the very high CRP group (1.81 [95% CI 1.14–2.88], *p* = 0.01; 1.74 [95% CI 1.18–2.57], *p* = 0.005, respectively). Relationships were not observed between CRP, neuropathic pain, and breakthrough pain.

***Conclusions:*** The results indicated direct relationships between CRP, pain NRS, and pain IPOS.

## Introduction

Accumulating evidence suggests that systemic inflammation and inflammation in the tumor microenvironment are some of the mechanisms underlying cancer cachexia.^1–6^ Pain and other symptoms, such as anorexia, fatigue, drowsiness, depression, anxiety, and delirium, have frequently been detected under conditions in which a systemic inflammatory response occurs in patients with advanced cancer.^7–14^ Furthermore, serum levels of C-reactive protein (CRP) have been identified as a surrogate of systemic inflammation related to survival, the activities of daily living, physical symptoms, and psychological symptoms.^7–14^ A large prospective cohort study reported that the positive rates of symptoms increased as CRP levels became higher.^11^ It also showed that the rates of positivity for anorexia and fatigue were 80%–90% in patients with very high CRP levels.^11^ Another prospective cohort study revealed that the incidence of drowsiness and delirium significantly increased as CRP levels became higher.^14^ In addition, two studies showed that CRP levels were related to pain with a comprehensive evaluation in advanced cancer,^7^,^8^ whereas the same group also reported a relationship between these two variables.^10^,^15^

Therefore, to the best of our knowledge, limited information is currently available on the relationship between elevated CRP levels and pain in patients with cancer cachexia. Furthermore, it has not yet been established whether relationships exist between elevated CRP levels and several subtypes of pain, including nociceptive pain, neuropathic pain, and breakthrough pain, even though pain is the most common symptom in patients with advanced cancer receiving palliative care.

Therefore, we conducted a secondary analysis of a prospective cohort study in palliative care units across Japan to investigate the relationships between serum levels of CRP and subtypes of pain in patients with advanced cancer cachexia. We also examined the current implementation of opioid medications for cancer pain among groups according to CRP levels because opioids may be a confounding factor in the relationship between CRP levels and pain.

## Materials and Methods

### Sites and participants

This study was a secondary cross-sectional analysis of a large multicenter prospective cohort study, which was conducted at 23 palliative care units in Japan between January 2017 and June 2018. In brief, consecutive patients who had been newly referred to these palliative care units were enrolled. All patients were followed up to their death or six months after enrollment. All institutions consecutively obtained a sample of data, up to the designated number of patients of 50, 60, 70, 80, 100, 150, and 250 according to the size of the institution. Inclusion criteria were (1) adult patients (18 years or older), (2) patients with locally advanced or metastatic cancer (including hematological neoplasms), and (3) patients admitted to palliative care units. Patients who planned to be discharged within one week or those who did not want to participate were excluded from this study.

This study was conducted in accordance with the ethical standards of the Declaration of Helsinki and ethical guidelines for epidemiological research presented by the Ministry of Health, Labour, and Welfare in Japan. The study was approved by the local institutional review boards of all participating institutions. Since Japanese law does not require individual informed consent from participants in a noninvasive observational trial, we used an opt-out method rather than acquiring written or oral informed consent.

### Measurements

Patient characteristics (age, gender, primary cancer site, the presence of metastasis, chemotherapy or targeted therapy within one month, and the Eastern Cooperative Oncology Group Performance Status [ECOG PS]^16^) were obtained at baseline.

Palliative care physicians asked patients to rate the severity of pain with a comprehensive evaluation on the numerical rating scale (NRS) ranging between 0 (not at all) and 10 (overwhelming), and physicians also evaluated the integrated palliative care outcome scale (IPOS)^17^ ranging between 0 (not at all) and 4 (overwhelming) at baseline. They assessed neuropathic pain and breakthrough pain according to the Clinical Guidelines for Cancer Pain Management edited by the Japanese Society for Palliative Medicine^18^ based on their presence or absence at baseline.

The types of opioids, administration routes of opioids, and opioid oral morphine milligram equivalent (mg/day) were recorded by palliative care physicians at baseline. Laboratory data (serum levels of albumin and CRP) measured within seven days before admission or three days after admission were also recorded. Survival was defined as the time from admission to a palliative care unit to death or discharge. Patients being discharged had been followed up for six months from baseline.

### Statistical analysis

Patient characteristics are shown as a mean ± standard deviation, median (interquartile range), or as a *n* (%) where appropriate. Patients were divided into four groups according to CRP levels: (1) low (CRP <1 mg/dL), (2) moderate (1 ≤ CRP <5 mg/dL), (3) high (5 ≤ CRP <10 mg/dL), and (4) very high (10 mg/dL ≤ CRP). We used approximate figures to quartile points, as described in our previous studies,^9^,^11^,^14^ which indicated the utility of CRP for predicting survival, the activities of daily living, physical symptoms, and psychological symptoms in patients with advanced cancer.

Comparisons among the groups were performed using the Kruskal–Wallis test or chi-squared test where appropriate. Spearman's correlation coefficients between pain NRS and pain IPOS and between CRP and pain were calculated to assess the relationship between two variables using a monotonic function; Spearman's correlation coefficient: <0.2 poor agreement, 0.21–0.4 fair, 0.41–0.6 moderate, 0.61–0.8 good, 0.81–0.99 very good, and 1 perfect.^19^

To evaluate the relationship between the four CRP groups, pain NRS (0–3 or 4–10),^20^ pain IPOS (0–1 or 2–4),^21^ neuropathic pain (presence or absence), and breakthrough pain (presence or absence), adjusted odds ratios (ORs), and 95% confidence intervals (CIs) were calculated after adjustments for independent variables known as potential risk factors for the development of pain in cancer patients, such as age, gender, metastasis, chemotherapy or targeted therapy within one month, ECOG PS, and the opioid oral morphine milligram equivalent.^7^,^8^,^10^,^12^,^15^

They were entered into the logistic model using the forced entry method. We sequentially introduced variables into the model. Demographic and biological variables were followed by the opioid dose to clarify the influence of opioids as a confounding factor on the effects of other factors for pain. All results were considered to be significant when the *p*-value was <0.05. All analyses were performed using SPSS software version 22.0 (SPSS, Inc., Chicago, IL).

## Results

Among the original cohort of 1896 patients, 383 were excluded due to missing data on main outcome variables, such as CRP, pain NRS, and pain IPOS. Therefore, 1513 patients (79.8%) were considered to be eligible for analyses.

Patient characteristics are summarized in Table 1. Mean age was 72.5 ± 12.2 years, and the percentage of male patients was 50.5%. The top three sites of primary cancer were the upper and lower gastrointestinal tract, the liver/biliary system/pancreas, and the lungs. The percentage of patients with metastasis was 84.9%. The percentage of patients receiving chemotherapy or targeted therapy within one month was 9.2%. The percentages of patients with ECOG PS 3 and 4 were 46.3% and 44.4%, respectively. Mean serum levels of albumin and CRP were 2.5 ± 0.7 g/dL and 7.5 ± 7.3 mg/dL, respectively. The median actual survival time was 20.0 (9.0–43.0) days.

**Table 1.** Patient Characteristics (*n* = 1513)

Age in years, mean (SD)	72.5 (12.2)
Gender, *n* (%)	
Male	764 (50.5)
Female	749 (49.5)
Primary cancer site, *n* (%)	
Upper and lower gastrointestinal tract	421 (27.8)
Liver, biliary system, and pancreas	294 (19.4)
Lung	243 (16.1)
Urological	115 (7.6)
Breast	108 (7.1)
Gynecological	94 (6.2)
Head and neck	62 (4.1)
Hematological	42 (2.8)
Others	134 (8.9)
Metastasis, yes, *n* (%)	1285 (84.9)
Chemotherapy or targeted therapy within one month, yes, *n* (%)	139 (9.2)
ECOG PS, *n* (%)	
0–1	20 (1.3)
2	121 (8.0)
3	700 (46.3)
4	672 (44.4)
Serum levels, mean (SD)	
Albumin (g/dL)	2.5 (0.7)
CRP (mg/dL)	7.5 (7.3)
Survival time (days), median (IQR)	20.0 (9.0–43.0)

Values are means ± SD, median (IQR), or *n* (%).

CRP, C-reactive protein; ECOG PS, Eastern Cooperative Oncology Group performance status; IQR, interquartile range; SD, standard deviation.

We then divided patients into four groups according to CRP levels: (1) low (CRP <1 mg/dL) (*n* = 234), (2) moderate (1 ≤ CRP <5 mg/dL) (*n* = 513), (3) high (5 ≤ CRP <10 mg/dL) (*n* = 352), and (4) very high (10 mg/dL ≤ CRP) (*n* = 414).

The current implementation of opioid medications for cancer pain is given in Table 2. The average percentage of patients reporting any opioid use was 65.2%, which increased as CRP levels became higher. Regarding the administration routes of opioids, a subcutaneous or intravenous route was the most frequent (33.1%) in all patients, and reached 44.0% with increases in CRP levels. The mean values of the opioid oral morphine milligram equivalent in all patients and in the high CRP group (5 ≤ CRP <10 mg/dL) were 45.5 ± 90.6 mg/day and 60.5 ± 134.2 mg/day, respectively. The mean value of the opioid oral morphine milligram equivalent in the high CRP group was the highest among the four groups categorized according to CRP levels (Table 2).

**Table 2.** Prevalence and Use of Opioids According to C-Reactive Protein Levels

	Total (*n* = 1513)	CRP <1 (*n* = 234)	1 ≤ CRP <5 (*n* = 513)	5 ≤ CRP <10 (*n* = 352)	10 ≤ CRP (*n* = 414)
Types of opioids, *n* (%)					
None	526 (34.8)	117 (50.0)	203 (39.6)	104 (29.5)	102 (24.6)
Morphine	291 (19.2)	36 (15.4)	88 (17.2)	71 (20.2)	96 (23.2)
Oxycodone	412 (27.2)	42 (17.9)	121 (23.6)	108 (30.7)	141 (34.1)
Fentanyl	244 (16.1)	32 (13.7)	85 (16.6)	56 (15.9)	71 (17.1)
Tramadol	44 (2.9)	7 (3.0)	17 (3.3)	10 (2.8)	10 (2.4)
Codeine, tapentadol, hydromorphone, or methadone	42 (2.8)	6 (2.6)	13 (2.5)	14 (4.0)	9 (2.2)
Administration routes of opioids, *n* (%)					
Oral	340 (22.5)	51 (21.8)	112 (21.8)	87 (24.7)	90 (21.7)
Patch	183 (12.1)	26 (11.1)	62 (12.1)	45 (12.8)	50 (12.1)
Subcutaneous or intravenous	500 (33.1)	46 (19.6)	143 (27.9)	129 (36.6)	182 (44.0)
Suppository or buccal	9 (0.6)	1 (0.4)	4 (0.8)	1 (0.3)	3 (0.7)
Opioid oral morphine milligram equivalent (mg/day), mean (SD)	45.5 (90.6)	24.8 (47.8)	41.3 (75.7)	60.5 (134.2)	49.5 (76.6)

The sums of some percentages were >100% because of the concurrent use of two or more types of opioids.

Spearman's correlation coefficient between pain NRS and pain IPOS was 0.66 (*p* < 0.001). The relationships between CRP and pain are given in Table 3. Spearman’s correlation coefficient between CRP and pain NRS and that between CRP and pain IPOS were 0.15 (*p* < 0.001) and 0.16 (*p* < 0.001), respectively. The proportions of pain NRS (4–10) and pain IPOS (2–4) and the positive rates of neuropathic pain and breakthrough pain in the four CRP groups are also summarized in Table 3. The proportions of pain NRS (4–10) and pain IPOS (2–4) significantly increased as CRP levels became higher (*p* < 0.001), whereas the positive rates of neuropathic pain and breakthrough pain did not significantly change.

**Table 3.** Relationships Between C-Reactive Protein Levels and Pain

	Total (*n* = 1513)	CRP <1 (*n* = 234)	1 ≤ CRP <5 (*n* = 513)	5 ≤ CRP <10 (*n* = 352)	10 ≤ CRP (*n* = 414)	*p*
Pain NRS, median (IQR)	2.0 (0.0–4.0)	1.0 (0.0–3.0)	1.0 (0.0–3.0)	2.0 (0.0–4.0)	2.0 (0.0–4.0)	<0.001
Spearman's correlation coefficient		0.15				<0.001
Pain NRS, 4–10, *n* (%)	320 (27.9)	39 (20.1)	88 (23.8)	78 (29.2)	115 (36.2)	<0.001
Pain IPOS, median (IQR)	1.0 (0.0–2.0)	1.0 (0.0–2.0)	1.0 (0.0–2.0)	1.0 (0.0–2.0)	1.0 (1.0–2.0)	<0.001
Spearman's correlation coefficient		0.16				<0.001
Pain IPOS, 2–4, *n* (%)	565 (37.4)	62 (26.5)	175 (34.1)	139 (39.5)	189 (45.8)	<0.001
Neuropathic pain, yes, *n* (%)	211 (14.0)	31 (13.4)	63 (12.3)	51 (14.5)	66 (15.9)	0.45
Breakthrough pain, yes, *n* (%)	601 (39.8)	82 (35.3)	191 (37.4)	145 (41.2)	183 (44.2)	0.08

Comparisons among groups were performed using the Kruskal–Wallis test or chi-squared test where appropriate.

IPOS, integrated palliative care outcome scale; NRS, numerical rating scale.

Adjusted ORs for CRP and other variables associated with pain NRS and pain IPOS are given in Tables 4 and 5, respectively. Six variables other than the opioid oral morphine milligram equivalent were included in model 1, whereas seven were included in model 2. In model 1 of pain NRS, there were significant differences in the adjusted ORs between the low CRP group and the high CRP and very high CRP groups (1.64 [95% CI 1.04–2.57], *p* = 0.03; 2.34 [95% CI 1.51–3.62], *p* < 0.001, respectively). In model 2 of pain NRS, there was a significant difference in the adjusted OR between the low CRP group and the very high CRP group (1.81 [95% CI 1.14–2.88], *p* = 0.01) (Table 4).

**Table 4.** Odds Ratios of Pain Numerical Rating Scale (4–10) by C-Reactive Protein Levels (*n* = 1148)

	Univariate analysis		Multivariate analysis			
			Model 1		Model 2	
	Crude OR (95% CI)	*p*	Adjusted OR (95% CI)	*p*	Adjusted OR (95% CI)	*p*
CRP (mg/dL)						
CRP <1	1.00 (reference)		1.00 (reference)		1.00 (reference)	
1 ≤ CRP <5	1.25 (0.81–1.90)	0.31	1.27 (0.82–1.95)	0.29	1.10 (0.70–1.74)	0.69
5 ≤ CRP <10	1.64 (1.06–2.55)	0.03	1.64 (1.04–2.57)	0.03	1.29 (0.80–2.07)	0.30
10 ≤ CRP	2.25 (1.48–3.42)	<0.001	2.34 (1.51–3.62)	<0.001	1.81 (1.14–2.88)	0.01
Opioids (mg/day)						
0	—	—	—	—	1.00 (reference)	
0–60	—	—	—	—	4.53 (3.02–6.81)	<0.001
60≤	—	—	—	—	9.11 (5.82–14.27)	<0.001

To evaluate the relationship between the four CRP groups and pain NRS (0–3 or 4–10), ORs and 95% CIs were calculated after adjustments for independent variables, such as age, gender, metastasis, chemotherapy, or targeted therapy within one month, the ECOG PS, and the opioid oral morphine milligram equivalent.

CI, confidence interval; OR, odds ratio.

**Table 5.** Odds Ratios of Pain Integrated Palliative Care Outcome Scale (2–4) by C-Reactive Protein Levels (*n* = 1512)

	Univariate analysis		Multivariate analysis			
			Model 1		Model 2	
	Crude OR (95% CI)	*p*	Adjusted OR (95% CI)	*p*	Adjusted OR (95% CI)	*p*
CRP (mg/dL)						
CRP <1	1.00 (reference)		1.00 (reference)		1.00 (reference)	
1 ≤ CRP <5	1.44 (1.02–2.02)	0.39	1.49 (1.05–2.12)	0.03	1.27 (0.87–1.85)	0.21
5 ≤ CRP <10	1.81 (1.26–2.60)	0.001	1.82 (1.26–2.64)	0.002	1.37 (0.92–2.03)	0.12
10 ≤ CRP	2.34 (1.65–3.32)	<0.001	2.31 (1.61–3.33)	<0.001	1.74 (1.18–2.57)	0.005
Opioids (mg/day)						
0	—	—	—	—	1.00 (reference)	
0–60	—	—	—	—	3.94 (2.92–5.31)	<0.001
60 ≤	—	—	—	—	9.33 (6.62–13.15)	<0.001

To evaluate the relationship between the four CRP groups and pain IPOS (0–1 or 2–4), ORs and 95% CIs were calculated after adjustments for independent variables, such as age, gender, metastasis, chemotherapy or targeted therapy within one month, the ECOG PS, and the opioid oral morphine milligram equivalent.

However, in model 1 of pain IPOS, there were significant differences in the adjusted ORs between the low CRP group and the moderate CRP, high CRP, and very high CRP groups (1.49 [95% CI 1.05–2.12], *p* = 0.03; 1.82 [95% CI 1.26–2.64], *p* = 0.002; 2.31 [95% CI 1.61–3.33], *p* < 0.001, respectively). In model 2 of pain IPOS, there was a significant difference in the adjusted OR between the low CRP group and the very high CRP group (1.74 [95% CI 1.18–2.57], *p* = 0.005) (Table 5).

Adjusted ORs for CRP and other variables associated with neuropathic pain and breakthrough pain are also given in Tables 6 and 7, respectively. No relationships were observed between CRP, neuropathic pain, and breakthrough pain.

**Table 6.** Odds Ratios of Neuropathic Pain by C-Reactive Protein Levels (*n* = 1509)

	Univariate analysis		Multivariate analysis			
			Model 1		Model 2	
	Crude OR (95% CI)	*p*	Adjusted OR (95% CI)	*p*	Adjusted OR (95% CI)	Adjusted OR (95% CI)
CRP (mg/dL)						
CRP <1	1.00 (reference)		1.00 (reference)		1.00 (reference)	
1 ≤ CRP <5	0.91 (0.58–1.45)	0.70	0.92 (0.58–1.47)	0.73	0.78 (0.48–1.26)	0.30
5 ≤ CRP <10	1.10 (0.68–1.78)	0.70	1.05 (0.64–1.72)	0.84	0.80 (0.49–1.33)	0.40
10 ≤ CRP	1.23 (0.78–1.95)	0.38	1.14 (0.71–1.83)	0.60	0.86 (0.52–1.40)	0.54
Opioids (mg/day)						
0	—	—	—	—	1.00 (reference)	
0–60	—	—	—	—	3.70 (2.30–5.93)	<0.001
60 ≤	—	—	—	—	6.43 (3.90–10.58)	<0.001

To evaluate the relationship between the four CRP groups and neuropathic pain (presence or absence), ORs and 95% CIs were calculated after adjustments for independent variables, such as age, gender, metastasis, chemotherapy or targeted therapy within one month, the ECOG PS, and the opioid oral morphine milligram equivalent.

**Table 7.** Odds Ratios of Breakthrough Pain by C-Reactive Protein Levels (*n* = 1509)

	Univariate analysis		Multivariate analysis			
			Model 1		Model 2	
	Crude OR (95% CI)	*p*	Adjusted OR (95% CI)	*p*	Adjusted OR (95% CI)	*p*
CRP (mg/dL)						
CRP <1	1.00 (reference)		1.00 (reference)		1.00 (reference)	
1 ≤ CRP <5	1.09 (0.79–1.51)	0.59	1.11 (0.80–1.55)	0.53	0.92 (0.65–1.31)	0.66
5 ≤ CRP <10	1.28 (0.91–1.81)	0.16	1.25 (0.88–1.77)	0.22	0.92 (0.63–1.33)	0.64
10 ≤ CRP	1.45 (1.04–2.02)	0.03	1.37 (0.97–1.93)	0.08	0.99 (0.69–1.44)	0.97
Opioids (mg/day)						
0	—	—	—	—	1.00 (reference)	
0–60	—	—	—	—	3.29 (2.49–4.35)	<0.001
60 ≤	—	—	—	—	7.47 (5.39–10.36)	<0.001

To evaluate the relationship between the four CRP groups and breakthrough pain (presence or absence), ORs and 95% CIs were calculated after adjustments for independent variables, such as age, gender, metastasis, chemotherapy or targeted therapy within one month, the ECOG PS, and the opioid oral morphine milligram equivalent.

## Discussion

To the best of our knowledge, this is the first large cross-sectional study to report relationships between CRP levels and pain, particularly subtypes of pain, in patients with advanced cancer admitted to palliative care units who had a survival time of days to weeks. The majority of patients in this study may have been at the stage of refractory cachexia from the viewpoint of survival, because the median actual survival time was 20.0 days.^2^ The results obtained demonstrated that pain positively correlated with CRP levels in this population.

The results of this study indicate that pain, except for neuropathic pain and breakthrough pain, was associated with CRP levels. Increases in CRP levels were associated with the worsening of pain NRS and pain IPOS. Nevertheless, it is important to note that pain NRS and pain IPOS may collectively comprise nociceptive pain, neuropathic pain, and breakthrough pain.

Although Spearman's correlation coefficients were weak and statistically significant in the context of the large sample size, correlations remained in logistic models. However, the influence of opioids as a confounding factor on the effects of other factors for pain cannot be ignored. A high CRP level (5 ≤ CRP <10 mg/dL) was significant in model 1, but not in model 2 for both pain NRS and pain IPOS.

However, we previously reported that high CRP levels were associated not only with poor survival and deteriorations in the activities of daily living, but also with impaired consciousness.^9^,^11^,^14^ This result suggests that patients categorized into the very high CRP group may be almost bedridden due to deteriorations in the activities of daily living and impaired consciousness and that pain may not be as severe as that in the high CRP group, which is supported by the dose of opioids in the high CRP group being the highest among the four groups examined.

After controlling for the confounding influence of opioids, a correlation was only observed between the very high CRP level (10 mg/dL ≤ CRP) and pain NRS and pain IPOS. This suggests a relationship with the threshold between CRP and pain. Furthermore, the present results are consistent with previous findings showing relationships between systemic inflammation measured using serum levels of CRP and pain in advanced cancer.^7^,^8^ However, both studies used a univariate correlation analysis and showed a very weak correlation between CRP and pain, which may have been due to the large sample size.

Relationships between CRP levels, neuropathic pain, and breakthrough pain were not observed in this study. Neuropathic pain is caused by direct damage to the nervous system from a primary tumor, metastases, or cancer treatment, such as chemotherapy. A nerve may be infiltrated or compressed by a tumor or strangulated by fibrosis.^22–26^ Inflammation in the tumor microenvironment may affect the surrounding nerves, but systemic inflammation is not always necessary in the genesis of neuropathic pain.

Moreover, neuropathic pain may have included chemotherapy-induced peripheral neuropathy and postherpetic neuralgia in this study, although the percentage of patients receiving chemotherapy or targeted therapy within one month was very small in this population. Furthermore, neuropathic pain has been associated with breakthrough pain.^22–26^ In this context, neither neuropathic nor breakthrough pain was associated with disease progression or serum levels of CRP.

Although the role of systemic inflammation in the genesis of multiple symptoms currently remains unclear, our previous findings and the present results indicate the clinical utility of high serum levels of CRP, for example, 10 mg/dL ≤ CRP, for predicting survival, the activities of daily living, physical symptoms, and psychological symptoms in patients with advanced cancer.^9^,^11^,^14^

Hence, therapies to control systemic inflammation, for example, corticosteroids, nonsteroidal anti-inflammatory drugs, nutrition interventions, exercise, and a good sleep at night, appear to be vital for the management of pain, fatigue, and other physical and psychological symptoms related to cancer cachexia.^27–30^ These therapies may also prolong survival and improve the activities of daily living in patients with advanced cancer cachexia in palliative care settings. Further research is warranted.

This study has several limitations that need to be addressed. Measurement errors may be large, because this was a multicenter study in which multiple physicians participated. However, we measured symptoms according to clinical guidelines^18^ to improve the quality of data. In addition, the results obtained did not demonstrate a causal relationship between CRP levels and pain. There may have been unmeasured confounding factors and reverse causality. Elevated CRP levels may be a consequence of pain. Hence, causality remains unclear due to the characteristics of an observational study.

Moreover, the very high CRP group (10 mg/dL ≤ CRP) may have had coexisting acute infections or medical conditions. Nevertheless, the clinical implications of CRP remain unchanged, because these factors also aggravate clinical outcomes and deteriorate physical and psychological symptoms. In addition, the results of this study were influenced slightly by cancer treatments, including chemotherapy and radiotherapy, causing systemic inflammation, because all subjects were admitted to palliative care units after the cessation of cancer treatments.

Another limitation is that the effects of medical agents suppressing systemic inflammation, such as corticosteroids and nonsteroidal anti-inflammatory drugs, and adjuvant analgesics in addition to opioids, were not considered. Furthermore, pain may have comprised nociceptive pain, neuropathic pain, mixed nociceptive–neuropathic pain, breakthrough pain, and even chronic pain from sources other than cancer in this study. However, a comprehensive evaluation of pain is necessary and useful for both patients and physicians to measure the quality of life of patients in daily clinical practice.

## Conclusions

The present results indicate that significant and direct relationships exist between CRP levels and pain in patients with advanced cancer cachexia. This study also describes the current implementation of opioid medication for cancer pain in palliative care units in Japan. Studies that target systemic inflammation and assess its impact on pain and other symptoms from the viewpoint of cancer cachexia are important. Further research is warranted in the near future.

## Data Availability

The data sets generated and/or analyzed during this study are not publicly available as sharing is not explicitly covered by patient consent.

## Notes

**Cite this article as:** Amano K, Ishiki H, Miura T, Maeda I, Hatano Y, Oyamada S, Yokomichi N, Tagami K, Odagiri T, Ito T, Baba M, Morita T, and Mori M; on behalf of the East-Asian Collaborative Cross-Cultural Study to Elucidate the Dying Process (EASED) Investigators (2021) C-reactive protein and its relationship with pain in patients with advanced cancer cachexia: Secondary cross-sectional analysis of a multicenter prospective cohort study, *Palliative Medicine Reports* 2:1, 122–131, DOI: 10.1089/pmr.2021.0004.

## Abbreviations Used

CI: confidence interval
CRP: C-reactive protein
ECOG PS: Eastern Cooperative Oncology Group Performance Status
IPOS: integrated palliative care outcome scale
IQR: interquartile range
NRS: numerical rating scale
OR: odds ratio
SD: standard deviation
